# Recent advances in the management of immune thrombocytopenic purpura (ITP): A comprehensive review

**DOI:** 10.1097/MD.0000000000036936

**Published:** 2024-01-19

**Authors:** Mohammed Ali Madkhali

**Affiliations:** a Department of Internal Medicine, Division of Hematology and Oncology, Faculty of Medicine, Jazan University, Jazan, Saudi Arabia.

**Keywords:** B and T cells, immune thrombocytopenic purpura (ITP), immunopathogenesis, severe bleeding, thrombocytopenia, thrombopoietin receptor agonists (TPO-RAs)

## Abstract

Autoimmune disorders place a substantial burden on the healthcare system all over the world affecting almost 3% to 8% of the population. Immune thrombocytopenic purpura (ITP), also known as idiopathic thrombocytopenic purpura, is a blood disorder in which the body immune system destroys platelets, leading to low platelet counts in the blood (peripheral blood platelet count < 150 × 109/L). Although the pathophysiology of ITP is not fully understood, it is believed to result from a complex interplay between hereditary and environmental variables. Certain factors, such as a low platelet count, history of bleeding, and certain comorbidities can increase the risk of severe bleeding in patients with ITP. Corticosteroids, intravenous immunoglobulin (IVIG), immunosuppressants, rituximab, and thrombopoietin receptor agonists (TPO-RAs) are some of the advanced treatments for ITP. Although these therapies may be successful, they also carry the risk of negative effects. Recently, significant advancements have been made in the understanding and treatment of ITP. There is still much to learn about the disease, and new, more effective treatments are needed. This comprehensive review offers a comprehensive assessment of recent advancements in ITP management, with a focus on active research projects, novel therapeutic targets, new treatment modalities, and areas of uncertainty and unmet needs. According to research, it is crucial to develop individualized treatment plans for ITP patients based on their age, platelet count, risk of bleeding, and comorbidities. The article also looks at how future developments in gene editing, bispecific antibody therapies, and cellular therapy may completely change the treatment of ITP.

## 1. Introduction

### 1.1. Definition

Immune thrombocytopenic purpura (ITP) is a blood disorder characterized by the immune-facilitated destruction of platelets by autoantibodies and insistently lowered platelets in the blood. Bleeding can occur more easily when there are too few platelets that play a key role in blood clotting.^[1]^ This disease was previously known as Werlhof disease. The British Society of Hematology guidelines defined this syndrome as “an autoimmune disorder that causes a low platelet count due to autoantibodies binding to platelet antigens.” This leads to premature destruction of platelets by the reticuloendothelial system, particularly in the spleen and sometimes in the liver.^[1,2]^ Patients may be asymptomatic at presentation or present with mild mucocutaneous or life-threatening bleeding.^[3]^ There is a 15% probability of severe bleeding involving hospitalization within 5 years of diagnosis of ITP, even though only 5% of patients are present with severe bleeding at diagnosis. Notably, the risk of severe bleeding in ITP is not uniform. Certain factors, such as a depleted platelet count, history of hemorrhage, and certain comorbidities can increase the risk of severe bleeding in ITP patients.^[4]^ Several drugs have been linked with the onset of ITP by triggering the production of autoantibodies (anti-platelets) in some people, including acetazolamide, aspirin, digitoxin, methyldopa, sulfamethazine, cephalothin, rifampin, phenytoin carbamazepine, meprobamate, and quinidine. The development of autoantibodies against platelets has been suggested as a major cause of ITP.^[5]^

### 1.2. Epidemiology

According to the most recent Thrombocytopenia International Working Group consensus, children (five cases per 100,000) and adults (three cases per 100,000) have different annual incidences of ITP.^[6,7]^ Overall, it is predicted that there are 2 to 7 ITP cases per 100,000 people per year, with 2 peaks occurring between the ages of 20 and 30 and 60 and with equal sex distribution, respectively.^[7,8]^ Individuals of all ages are more susceptible to ITP.^[9]^

### 1.3. Classification

Immune thrombocytopenic purpura (ITP) can be classified as acute or chronic based on several factors, including the duration of the condition and its etiology. The acute type typically appears in children of both sexes and is characterized by a sudden onset of thrombocytopenia. It often follows a viral infection and spontaneously resolves within 3 to 5 months (85% cases), without the need for long-term treatment.^[10]^ The Chronic ITP affects people between 20 and 50 years of age and **i**s diagnosed when the condition persists for more than 6 months. It is not caused by viral infection. Unlike acute ITP, chronic ITP often needs continuing treatment and management to maintain safe platelet levels.^[5,10]^ According to international guidelines, ITP can be classified into primary and secondary ITP. Primary ITP can be defined as an acquired autoimmune disorder, characterized by increased humoral platelet destruction and/or impaired platelet production. These events are thought to be due to autoantibodies that target platelet external glycoproteins such as GPIb/IX and GPIIb/IIIa.^[10,11]^ Secondary ITP is caused by other conditions, such as autoimmune disease, infection, cancer, or medication.^[11]^ Specific autoimmune diseases include lymphoproliferative diseases, immunodeficiency diseases, and chronic infections,^[12]^ and certain drugs can also cause secondary ITP.^[13,14]^ Moreover, COVID infection was reported as a potential secondary cause of ITP.^[15–17]^

**Figure 1.**
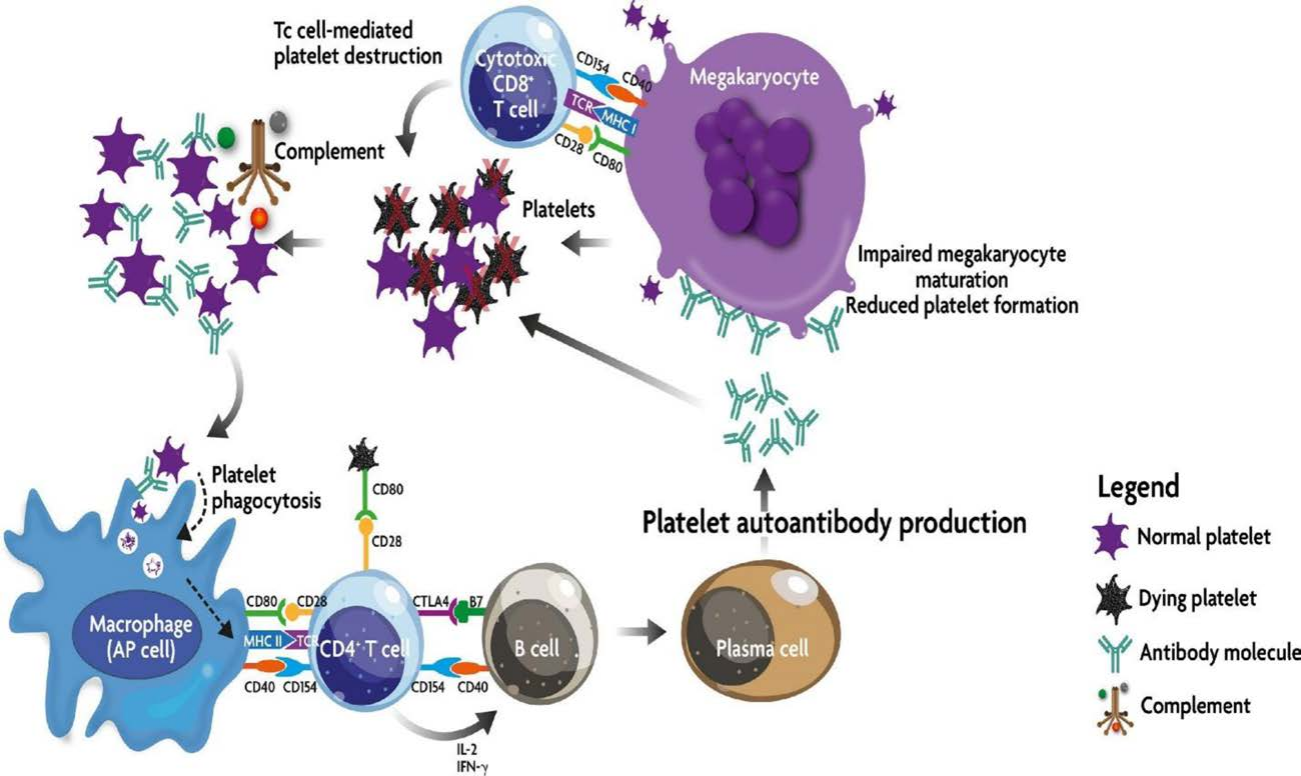
ITP immune effector mechanism: In ITP, bone marrow-based antigen-presenting cells (APCs) process and deliver platelet autoantigens to autoreactive T cells. This starts a chain of events that result in the creation of platelet-specific autoantibodies and the activation of cytotoxic T cells. In peripheral circulation, platelets can be destroyed by both autoantibodies and cytotoxic T lymphocytes. The complement cascade may also assault autoantibody-opsonized platelets. Autoantibodies and cytotoxic T lymphocytes can also prevent megakaryocytes in the bone marrow from producing platelets.^[5]^ *Source*: Provan D, Semple JW. Recent advances in the mechanism and treatment of immune thrombocytopenia. EBioMedicine, 2022;76:103820. CC BY-NC-ND 4.0.

With a focus on ongoing research projects, innovative therapeutic targets, new treatment methods, and areas of ambiguity and unmet needs, this thorough review provides a complete assessment of recent improvements in the management of ITP. Personalized treatment strategies for ITP patients must be developed based on factors such as age, platelet count, risk of bleeding, and comorbidities (Fig. 1).

## 2. Immunopathogenesis in the development of ITP

The immunopathogenesis of ITP is complex and involves several different immune cell types and mechanisms. The exact immunological mechanisms involved in the development of ITP are not fully understood; however, they are thought to involve a complex interplay between autoantibodies, reduced platelet production, T cells, B cells, and dysregulation of the immune system.

### 2.1. Autoantibody-mediated platelet destruction

Autoantibodies are directed against the body own tissues or cells. In ITP, autoantibodies produce antiplatelet surface glycoproteins, such as GPIb/IX and GPIIb/IIIa. Moreover, some patients with ITP have been reported to have anti-GPIa/IIa and anti-GPVI, which may affect the site of platelet destruction.^[18,19]^

These antibodies can impair megakaryocyte function and cause platelet death mediated by complement or desialylation.^[9–11]^ Antiplatelet antibodies are reported in most patients with ITP and are not detected in up to 50% of cases. This suggests that there may be other mechanisms of platelet destruction in ITP patients. One possible mechanism for this is T cell-mediated cytotoxicity. White blood cells (T cells) function in the immune system. T lymphocytes may overreact with target platelets in ITP. Impairment of the function of regulatory T cells is an additional potential mechanism of platelet lysis in ITP. Regulatory T cells help suppress the immune system. In ITP, the quantity and function of regulatory T cells may be reduced, leading to an overactive immune system and destruction of platelets.^[9,13]^ Limited studies also suggest that CD8 cells, another type of white blood cells, may be involved in the destruction of platelets in ITP.^[14]^

Role of T cells and B cells mechanism in ITP: In ITP, there is an observed increase in the activation and release of cytokines by Th1 and Th17 helper T cells. Th1 cells release interferon-γ (IFN-γ), which stimulates and amplifies the presence of immune cells, such as cytotoxic T lymphocytes, natural killer cells, and macrophages. This heightened immune activity can lead to cytotoxic effects and potentially affect the platelets. On the other hand, Th17 cells predominantly release interleukin-17 (IL-17), which can enhance the production of inflammatory signaling molecules and chemokines by various cell types. IL-17 recruit neutrophils and contributes to the initiation and progression of inflammatory responses. Th1 and Th17 cells are significant players in the autoimmune response observed in ITP, with Th1 cells showing promising immune cell proliferation that may target platelets, and Th17 cells fostering inflammation and neutrophil recruitment.^[20]^

Regarding the role of B cells in the development of ITP, immunoglobulin (IgG) antibodies that interact with platelets are the main causes of ITP.^[21]^ These antibodies bind to platelets, effectively marking them as phagocytic cells in the liver and spleen that remove them. This mechanism is known as antibody-mediated platelet destruction. IgG antibodies against platelet glycoproteins, with an emphasis on GPIIb/IIIa and GPIbIX, are the most common types and GPIa/IIA and GPVI are the less common types of antibodies observed in ITP patients.^[18–20]^ These antibodies bind to Fc receptors on macrophages, phagocytic cells that engulf and destroy foreign particles. Platelet clearance may be more important than decreased platelet production in ITP patients. However, some ITP patients also have cytotoxic CD8 + T cells, which can destroy megakaryocytes, the bone marrow cells that generate platelets.^[20,21]^

**Figure 2.**
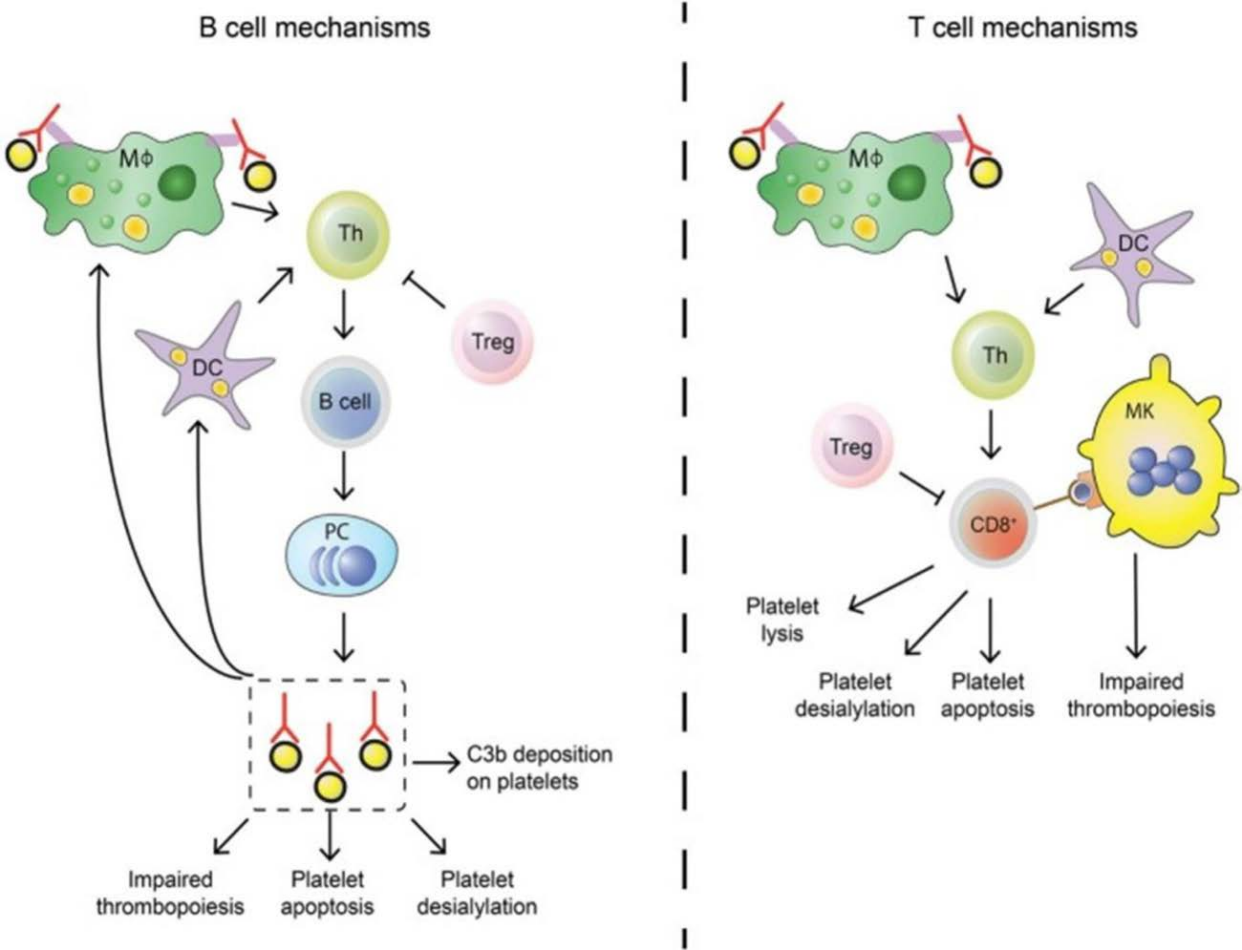
T cell and B cell mechanisms in pathogenesis of ITP: Both T cells and B cells play crucial roles in the development of ITP. Autoantibodies produced by B cells have the potential to impair platelet function and formation. In addition, platelets can be directly destroyed by cytotoxic T lymphocytes. Regulating the autoimmune response in ITP patients is frequently insufficient due to an imbalance in the numbers of regulatory T cells (Tregs)^[22]^ *Source*: Swinkels M, Rijkers M, Voorberg J, Vidarsson G, Leebeek FWG, Jansen AJG. Emerging Concepts in Immune Thrombocytopenia. Front Immunol, 2018 Apr 30;9:880. CC BY-NC-ND 4.0.

The spleen is an immune organ that participates in platelet destruction. Autoantibody-coated platelets are sequestered and destroyed in the spleen. Additionally, spleen may be a site of autoantibody production and T cell activation. Complex interactions between platelets, immunological cells, and the spleen are crucial for the emergence of ITP. Platelets can activate the immune cells and promote inflammation. Immune cells activate platelets and promote their destruction. Current research is focused on developing therapies that target the connections between the spleen, platelets, and immune cells in ITP patients. For example, some researchers have developed drugs that block the activation of platelets or immune cells. Other researchers are developing drugs that block the destruction of platelets in the spleen.^[6,20,21]^ Figure 2^[22]^.

## 3. Clinical manifestation and complications

### 3.1. Clinical manifestation

The clinical signs and symptoms of ITP can change depending on the severity of the platelet count and presence of any underlying medical disorders. The most common symptoms include purpura, bruising, nosebleeds, bleeding gums, petechiae, and heavy menstrual bleeding. In rare cases, ITP can cause severe bleeding, such as bleeding into the brain or abdomen. It is a medical emergency that requires immediate treatment. ITP must be diagnosed when the platelet count is <100,000/L.^[23]^ Notably, a sizeable percentage of patients could not show any obvious symptoms. However, up to 2-thirds of ITP patients may experience bleeding problems. When platelet counts fall below 20,000 to 30,000/L, bleeding is most likely to occur.^[23]^ Bleeding episodes frequently have a distinctive “platelet-type” pattern, which causes petechiae, bruises, epistaxis (nosebleeds), and gum bleeding. Moreover, “wet purpura,” characterized by hemorrhagic blisters on mucosal membranes, particularly in the oral cavity, might be a sign of more serious hemorrhagic episodes.^[24,25]^ Several databases, including patient registries, administrative databases, and pooled studies of clinical trials, have been used to estimate the rate of bleeding in ITP. Based on observational registry data, the Intercontinental Cooperative Immune Thrombocytopenia Study Group (ICIS) found that 24% of children and 23% of adults with newly diagnosed acute ITP showed indications of bleeding. Ten out of 1784 (0.6%) children and 6 out of 340 (1.8%) adults experienced intracranial hemorrhage.^[26]^ According to a thorough retrospective cohort study carried out in the United States, 57% of patients with chronic ITP experienced bleeding-related events during 13,064 patient-years of follow-up, with cerebral hemorrhage occurring in 1% of cases. Furthermore, a yearly cerebral hemorrhage rate of 0.4% in children and 1.8% in adults with ITP was calculated through a comprehensive evaluation of 118 papers.^[27]^

### 3.2. Complications associated with ITP

Although relatively uncommon, severe bleeding symptoms such as cerebral hemorrhage, gastrointestinal or genitourinary bleeding, or heavy menstrual bleeding are the main reasons for treating ITP. The risk of bleeding is directly linked to most ITP problems in both children and adults, particularly when the platelet count drops below 20,000/µL. It is vital to remember that most ITP sufferers exhibit symptoms such as bruises and petechiae.^[28]^ Some patients with ITP may experience mucosal bleeding such as gum bleeding or epistaxis. In extreme circumstances, the digestive tract may bleed, resulting in heme-positive feces, hematuria, or menorrhagia.^[29]^

Intracranial hemorrhage (ICH) is the most common complication of ITP. The risk of ICH is approximately 0.5% in children with freshly diagnosed ITP, and it is somewhat higher in children with chronic ITP, but still <1%. It is important to remember that most cases of ICH occur when platelet counts are lower than 10,000/microL.^[30]^ Headache, recurrent vomiting, altered mental status, seizures, focal neurological abnormalities, and/or a history of recent head trauma are symptoms that may cause ICH in both children and adults. In such circumstances, it is necessary to promptly assess the patient, which may entail neuroimaging and emergency care [25]. Extremely low platelet counts (< 10,000/µL), head injury, use of antiplatelet medicines, and severe bleeding are risk factors associated with an increased risk of ICH. Epistaxis lasting 5 to 15 minutes, gastrointestinal bleeding, or any other type of severe mucosal bleeding requiring hospitalization or blood transfusion are all considered serious bleeding.^[30]^ A comprehensive patient history is necessary to detect probable secondary ITP causes and illnesses that might only be blamed for thrombocytopenia, such as liver disease. Secondary ITP can be associated with autoimmune, immunodeficient, lymphoproliferative, and infectious conditions. It is important to diagnose secondary ITP because its treatment differs from that of primary ITP. Liver illness is frequently associated with thrombocytopenia. Numerous factors can contribute to this, such as reduced thrombopoietin in severe liver illness, sequestration caused by hypersplenism, or direct bone marrow suppression caused by alcohol toxicity.^[31]^

## 4. Diagnostic criteria

Low platelet count is a hallmark of ITP, a condition that may cause potentially fatal hemorrhage. To ensure proper treatment, it is crucial to correctly diagnose and comprehend the underlying cause of ITP. Comprehensive testing is necessary to eliminate all other probable reasons for a low platelet count because ITP is a diagnosis of exclusion. This evaluation also includes checking for diseases, such as HIV and HCV.^[32]^ noninvasive diagnostic procedures for ITP include stool antigen assays, anti-HP antibody serological tests, and urea breath tests. Endoscopy and biopsy may be required for invasive procedures, and the urease test is performed on the biopsy specimen. It is important to note that until prior therapies fail to provide beneficial results, culture and sensitivity testing are typically not advised.^[33]^ If there is a suspicion of an underlying immunodeficiency, both children and adults should receive a complete blood count with differential reticulocyte count, peripheral blood smear, blood type, direct antiglobulin test (DAT), and immunoglobulin levels. The only variance found in ITP is a platelet count of <100,000/microL.^[26]^ The WBC count, Hb concentration, RBC indices, and differential values were typically normal in this condition. Microcytic anemia can be found in complete blood count if there has been significant blood loss. Depending on the etiology of a recent infection in a patient, the white cell count can be high or low.^[30]^ On a peripheral blood smear from a patient with ITP, WBCs and RBCs typically appear normal, but the platelet count is decreased.^[34]^ A different underlying cause of thrombocytopenia may be revealed if further abnormalities are found in a peripheral blood smear. For instance, the presence of immature white blood cells (blasts) may indicate that a low platelet count is caused by diseases such as leukemia or lymphoma. Hemolytic anemia may be the underlying cause of thrombocytopenia when polychromasia, reticulocytes, or spherocytes are observed. Schistocyte development may indicate that microangiopathic hemolytic anemia is the primary cause of thrombocytopenia.^[35]^ Therefore, if a patient requires a blood transfusion, it is standard practice to check their blood type and perform a direct antiglobulin test (DAT) to determine if they have any antibodies. It is noteworthy that DAT test findings were typically unfavorable.

Histopathology of ITP commonly reveals enhanced megakaryocyte formation in the bone marrow. These findings suggest that decreased platelet production, rather than increased platelet breakdown, is the primary cause of thrombocytopenia.^[36]^ Bone marrow biopsy is not routinely performed in adults or children with ITP. It is recommended that patients not respond to treatment with glucocorticoids, intravenous immunoglobulin (IVIG), and/or anti-D immune globulin. Clinical or laboratory signs of malignancy or bone marrow failure (such as lymph node enlargement, splenomegaly, bone pain, fever, neutropenia, leukocytosis, atypical lymphocytes, or anemia) are also recommended. Bone marrow biopsy is only recommended for patients with ITP who have certain clinical features or who have not responded to other treatments.^[37]^

Antiplatelet autoantibody testing in primary ITP is highly specific but not very sensitive. This means that a positive test result strongly suggests ITP, whereas a negative test result does not rule out ITP. There are several possible reasons for the lower sensitivity of these tests. One possibility is that not all ITP patients have anti-GPIIb/IIIa or anti-GPIbIX autoantibodies. It is important to remember that alternate mechanisms, such as autoreactive CD8 + T cells, could be responsible for thrombocytopenia in patients lacking autoantibodies.^[38]^ Although antiplatelet autoantibody testing is the fundamental method for determining the existence of ITP, the comparatively low sensitivity of these tests implies that ITP can be caused by more than just the presence of antiplatelet autoantibodies.

A valuable approach for identifying problems in platelet function may be to examine platelet reactivity using flow cytometry. Small samples of whole blood can be used for this quick and accurate test even in patients with ICT.^[39]^ However, clinical trials must be conducted to validate flow cytometry before it can be used as a standard diagnostic technique. Since there is no test that can be used to diagnose problems in platelet function, this presents a problem. Researchers must find a sample of patients in which the existence or absence of the condition can be consistently confirmed or ruled out using a recognized gold standard test to validate a new platelet function test, such as flow cytometry.^[40]^

The ability to diagnose ITP using genetic testing has great potential. It can identify gene alterations that affect platelet function or generation, thus providing information about many facets of the illness. The identification of inherited thrombocytopenia syndromes, a rare group of illnesses characterized by low platelet counts, relies heavily on genetic testing. Currently, 329 known genes are associated with platelet function and thrombopoiesis, and it is anticipated that this number will increase with time.^[41]^ Additionally, genetic testing can help identify ITP individuals who may be at risk of serious bleeding or who are not likely to react to standard therapies. It is important to keep in mind that adult-onset hereditary thrombocytopenia syndromes can resemble recently diagnosed ITP, making access to genetic testing even more essential to ensure proper diagnosis and prevent unnecessary therapy.^[42]^ In the future, personalized treatment strategies for ITP patients may be developed using genetic testing. For instance, patients with mutations in specific genes may be treated with targeted medicines that aim to tackle the underlying cause of their condition.

## 5. Management strategies

ITP is characterized by several immune-related diseases, both humoral (linked to antibodies) and cellular, that collectively accelerate platelet destruction and impede platelet production. The main step in determining the presence of ITP is to rule out other causes of thrombocytopenia. It is vital to remember that corticosteroids, intravenous immunoglobulin (IVIG), splenectomy, and anti-RhD immunoglobulin have all been used in the past to treat ITP.^[28]^ The fact that these treatments have not been thoroughly investigated in randomized clinical trials must be emphasized, as these older therapies have variable efficacy and well-described side effects, with minimal benefits to many patients. Although they are used as the classical initial line of treatment for newly diagnosed ITP in adults, they frequently lose effectiveness over time. Weight gain, osteoporosis, and diabetes are some of the negative effects of corticosteroids.^[43]^ In 2020, a study was conducted on pregnant women to determine the effects of corticosteroids and IVIG on ITP. This study highlights that although immunoglobulins and corticosteroids are the first line of treatment for ITP, they have severe side effects on pregnant women even after delivery, such as postpartum hemorrhage, as compared to the second-line treatment of thrombocytopenic purpura.^[44]^

In the past 10 years, there has been a shift toward using treatments with reduced or no immune suppression, such as thrombopoietin receptor agonists (TPO-RAs). This shift has been accelerated by the COVID-19 pandemic, making it more important to develop non-immunosuppressive strategies for ITP. TPO-RAs are a promising new class of drugs that have been shown to be effective in increasing platelet counts and reducing bleeding in ITP patients. TPO-RAs are generally well tolerated and have a lower risk of side effects than older treatments. The shift away from immune suppression and toward TPO-RAs is a positive development in patients with ITP. TPO-RAs offer a safer and more effective way to manage this condition.^[45]^ In a study conducted in 2020, a thrombopoietin receptor agonist (TPO-RA) was approved for the treatment of immune thrombocytopenia. The number of patients was studied for a short period of time with a therapy of (TPO-RA) and it was concluded that they have a significant effect on ITP and are safe compared to surgical options.^[46]^

Under the leadership of Dr Tomás José González-López since September 2021, the Spanish ITP Group (GEPTI) has advised against the use of immunosuppressants, including rituximab, during the COVID-19 pandemic. This precaution is rooted in the heightened risk of severe SARS-CoV-2 infection associated with these medications.^[47]^ Prior to the pandemic, Spanish guidelines recommended these immunosuppressants as third-line treatment options for ITP, followed by corticosteroids, intravenous immunoglobulin, thrombopoietin receptor agonists (TPO-RAs), and Syk inhibitors. The GEPTI advises against the use of immunosuppressants for ITP during the COVID pandemic but recommends them as a third-line treatment option outside the pandemic.^[48]^ The 2 recently released ITP medications, fostamatinib and avatrombopag, are less expensive than the 2 TPO-RAs, romiplostim and eltrombopag, which are available in the market for a long period of time. For public health systems, such as Salud CastillayLeón (SACYL) in Spain, this represents considerable financial relief. In other words, the 2 more recent ITP medications are more reasonably priced, which is important for the public health system.

Individualized treatment approaches are crucial for ITP patients because ITP can manifest differently. While some patients experience minor symptoms with no bleeding, others experience major bleeding episodes that may be fatal. The way the disease progresses can also differ, with some individuals experiencing spontaneous remission and others developing chronic ITP that requires continuous care.^[49]^ Some patient characteristics are important to consider when developing an individualized treatment plan for ITP, such as age, platelet count, bleeding risk, and comorbidities (diabetes or cancer), which may be at higher risk for side effects from ITP treatment.^[50]^ Doctors can create individualized treatment strategies that are most likely to be efficient and secure for each patient by considering these aspects.

Treatment choices can be significantly influenced by patient characteristics, such as IVIG, and corticosteroids are frequently used in the treatment of ITP in children. Most children respond well to these medications and have a generally favorable safety profile. However, the negative effects of these medications may be more common in older ITP patients. As a result, medical professionals may be more inclined to suggest alternative therapies for elderly patients, such as thrombopoietin receptor agonists (TPO-RAs) or rituximab. Patients with extremely low platelet counts are at risk of potentially fatal hemorrhage.^[44,51]^ Doctors may advise on platelet transfusions under certain circumstances. However, platelet transfusions may have unfavorable side effects and are not always successful. As a result, for individuals with extremely low platelet counts, doctors may additionally suggest alternative therapies, such as TPO-RAs or rituximab. Patients are more likely to experience hemorrhage if they have a specific condition, such as liver or kidney disease. Clinicians may be more likely to recommend treatment to increase platelet counts in these circumstances, even when the patient platelet count is not severely low. Patients with conditions such as diabetes or cancer may experience ITP adverse effects of ITP more frequently. Therefore, medical professionals might need to alter the medication dosage or decide to adopt a different approach.^[46,52]^

## 6. Future directions and research

Researchers are investigating how specific immune cell types, such as T cells, B cells, and macrophages, contribute to the onset and progression of ITP. The creation of fresh and focused treatments may have resulted from this study. Although the precise genes involved remain unknown, genetics is thought to play a role in ITP. Additionally, scientists are researching how ITP is affected by epigenetics, which involves the study of how gene expression is controlled. This study may lead to the discovery of novel biomarkers for diagnosis and prognosis as well as new therapeutic targets. The gut microbiome is a group of microbes that inhabits the gut. The function of gut microbiota in the onset and progression of ITP has been studied by researchers. New noninvasive therapeutic approaches may have resulted from this research.

ITP therapies can be created using gene editing technologies such as CRISPR-Cas9. For instance, gene editing can be used to fix genetic changes linked to ITP or to knock off genes crucial to the maturation and operation of autoreactive immune cells. A novel class of medications called bispecific antibody treatments can simultaneously target 2 distinct molecules. New medicines for ITP that target both autoreactive immune cells and platelets may be created using this kind of therapy. The use of cells to cure diseases is a treatment method known as cellular therapy. Cellular therapies for ITP have been studied by researchers. For instance, scientists are developing CAR T cell treatments that specifically target autoreactive immune cells.

Currently available ITP therapies have mixed records of success and negative effects. Researchers have developed new therapies that are less harmful and more effective. To identify ITP patients who are most likely to experience severe bleeding, researchers are working to create new biomarkers. Individualized treatment programs for patients can be created using these data. ITP frequently enters remission after treatment; however, this condition is reversible. Researchers are developing novel therapies to prevent ITP from recurring.

In conclusion, tremendous progress has been made in understanding and managing ITP. The disease must still be fully understood, and new and efficient treatments are needed. The objective of continuing research is to address areas of uncertainty and unmet needs in the field, to better understand the pathophysiology and heterogeneity of ITP, to develop novel therapeutic targets and treatment modalities, and to better comprehend the heterogeneity of ITP. It is envisaged that, in the years to come, new and more effective treatments will be initiated because of the hopeful future of ITP research.

## Author contributions

**Conceptualization:** Mohammed Ali Madkhali.

**Data curation:** Mohammed Ali Madkhali.

**Formal analysis:** Mohammed Ali Madkhali.

**Methodology:** Mohammed Ali Madkhali.

**Supervision:** Mohammed Ali Madkhali.

**Writing – original draft:** Mohammed Ali Madkhali.

**Writing – review & editing:** Mohammed Ali Madkhali.
